# Language, Communicative Participation, and Well‐Being in Young Children with (Presumed) Developmental Language Disorder

**DOI:** 10.1111/1460-6984.70037

**Published:** 2025-04-23

**Authors:** Iris Duinmeijer, Sanne Peet, Lonneke Janssen, Annette Scheper, Margo Zwitserlood‐Nijenhuis, Wendy Bliekendaal, Marijke Zoons, Britt Hakvoort

**Affiliations:** ^1^ Research and Development Department NSDSK Amsterdam the Netherlands; ^2^ Department of Tranzo, Tilburg School of Social and Behavioral Sciences Tilburg University Amsterdam the Netherlands; ^3^ Research Department Royal Kentalis Utrecht the Netherlands; ^4^ Behavioural Science Institute Radboud University Nijmegen the Netherlands; ^5^ Pento Centre for Audiology Amersfoort Amersfoort the Netherlands; ^6^ Research Department Royal Dutch Auris Group Rotterdam the Netherlands; ^7^ Research Institute of Child Development and Education University of Amsterdam Amsterdam the Netherlands; ^8^ Adelante Centre for Rehabilitation & Audiology Hoensbroek the Netherlands

**Keywords:** communication, childcare, developmental language disorder, emotional health, longitudinal studies, quality of life, social behaviour

## Abstract

**Background:**

Children with developmental language disorder (DLD) have problems acquiring language, affecting their communicative participation, social–emotional functioning (SEF) and quality of life (QoL).

**Aims:**

To investigate whether communicative participation mediates the relation between language and SEF and QoL.

**Methods & Procedures:**

In a longitudinal design, 511 children were recruited via early intervention groups for children with (presumed) DLD. Language and IQ scores were obtained at a mean age of 3;11 (T0). In kindergarten, communicative participation, SEF and QoL were measured via parental questionnaires (T1, mean age 4;8). The relationship between language and SEF and QoL was investigated directly and with communicative participation as a mediating factor using structural equation modelling.

**Outcomes & Results:**

Expressive grammar was related to communicative participation, SEF and QoL, while receptive language and expressive vocabulary were not. Children with better expressive grammar at T0 showed better communicative participation at T1. Better communicative participation, in turn, was related to less problems in SEF and higher QoL. We also found an unexpected positive direct relation between expressive grammar and problems in SEF. Post‐hoc analyses showed that this was likely to be a suppressor effect, caused by a small subset of children with relatively good expressive grammar and poor communicative participation.

**Conclusions & Implications:**

Communicative participation is a mediator in the relation between language and SEF and QoL. These results underline the importance of addressing communicative participation as a functional measure of language ability both in research and clinical practice.

**WHAT THIS PAPER ADDS:**

*What is already known on the subject*

- Children with DLD have problems acquiring language and communication skills. Alongside and related to these challenges, many children with DLD experience greater problems in SEF and lower levels of QoL, although there is considerable variation among children. Previous research has demonstrated that structural language abilities only explain a small part of the variance in well‐being, and more functional language measures, such as pragmatic skills, play an important role.

*What this paper adds to the existing knowledge*

- This study investigated whether the relation between language problems and well‐being is mediated by children's ability to participate in communication (communicative participation). Structural equation modelling in a large, longitudinal sample of children with (presumed) DLD showed that communicative participation mediates the relationship between language abilities and both SEF and QoL. Better expressive grammatical skills were associated with better communicative participation, which in turn was related to higher SEF and QoL scores. Thus, the relation between language and well‐being is mediated by how effectively children can communicate in daily life.

*What are the potential or actual clinical implications of this work?*

- For clinicians, this study underlines the importance of addressing communicative participation as a functional measure of language ability. Measuring communicative participation can aid in identifying a child's specific needs and determining the most suitable setting for providing support. Measuring communicative participation can also assist clinicians in setting treatment goals, evaluating intervention effects, and providing advice to parents and schools. Furthermore, this study underscores the importance of recognizing that early language difficulties can affect well‐being.

## 1 Introduction

Approximately 5% of all young Dutch children have a developmental language disorder (DLD; Reep‐van den Bergh et al. 1998). This means they have difficulties acquiring language that are not explained by impairments in hearing, motor skills, neurological conditions or cognition (Bishop et al. 2016). The disorder manifests itself in difficulties to understand and/or use language in communicative contexts, but also has a clear impact on cognitive and academic development and on social and emotional well‐being as children grow older (Beitchman et al. 1996; Durkin and Conti‐Ramsden 2010). Already in the preschool years, children with speech and language difficulties are shown to have problems in connecting with peers and in regulating emotions (Andrés‐Roqueta et al. 2016; Chen et al. 2020; Gertner et al. 1994). Unsurprisingly, parents of children with DLD report more withdrawn and aggressive behaviour in their children, more signs of anxiety and depression in the preschool years, and more social problems in their first years in primary school (Maggio et al. 2014). These problems in social–emotional functioning (SEF) seem to increase with age (Curtis et al. 2018; Goh et al. 2021; Yew and O'Kearney 2013) and persist into adolescence (Van den Bedem, Dockrell, van Alphen, Samson et al. et al. 2018; Van den Bedem, Dockrell, van Alphen, Kalicharan, and Rieffe 2018).

Moreover, studies show that poor language abilities are related to the quality of life (QoL) of children and adolescents with DLD (Feeney et al. 2017; Le et al. 2020). QoL is a complex construct that denotes an individuals’ sense of well‐being. It encompasses aspects such as physical and psychological well‐being, school functioning, and relations with family or friends. Children with DLD seem to have a lower QoL compared with typically developing children and in contrast to peers, their QoL seems to decrease with age (Eadie et al. 2018). Low language skills are associated with poorer QoL from early childhood to adolescence (Le et al. 2021).

Although many children with DLD show difficulties in SEF and QoL, there also is considerable variation among children in outcomes on these domains. Interestingly, language abilities only explain part of the variation in SEF and QoL between children (Eadie et al. 2018; Goh et al. 2021). A potential other explanatory factor that has gained increasing attention in research and in clinical practice is communicative participation. Communicative participation can be defined as ‘understanding and being understood in a social context, by applying verbal and nonverbal communication skills’ (Singer et al. 2020: 1801). As such, communicative participation is considered a functional aspect of language ability, reflecting the (in)ability to use language in interaction with others.

In accordance with the International Classification of Functioning, Disability and Health (ICF) model (WHO 2001), information on the functional impact of language difficulties is deemed essential for identifying a child's needs (Bishop et al. 2016). Standardized measures of structural language abilities, such as a receptive vocabulary task or a grammatical inflection test, do not necessarily reflect the ability to use language in interaction. Across populations with speech, language, and communication disorders, weak correlations have been found between development in communicative participation and measures of speech abilities and expressive vocabulary (Cunningham et al. 2019, 2020). Monitoring communicative participation next to standardized language tests seems essential to be able to draw conclusions on the impact of language difficulties on everyday life.

Although communicative participation is increasingly measured in research and clinical practice to monitor treatment effects and to measure functional impairments in DLD, the relation between communicative participation and other developmental domains such as SEF and QoL in children with DLD is still unclear. In other clinical groups, communicative participation has been shown to be linked to well‐being outcomes. For instance, in adults with motor speech and voice disorders, the level of communicative participation appeared to be predictive for mental health (Jin et al. 2021). In a scoping review, Singer et al. (2022) also conclude that communicative participation is related to SEF factors such as prosocial behaviour, socio‐cognitive skills, and behavioural problems in children with DLD.

Communicative participation overlaps with pragmatic language skills (Singer et al. 2022). To be able to participate and communicate in social situations, pragmatic competence—for example, taking turns, adjusting language to the listener's knowledge, and understanding implicit information from a linguistic context—is pivotal. Communicative participation itself is often addressed using questionnaires that also tap into these pragmatic aspects (e.g., the FOCUS‐34 contains questions such as ‘my child needs help to be understood by other children’; Thomas‐Stonell et al. 2015).

Several studies indicate relations between pragmatics and SEF (e.g., Conti‐Ramsden et al. 2019; St. Clair et al. 2011). For instance, more problems in pragmatics lead to the use of more externalizing strategies in children with DLD (Van den Bedem, Dockrell, van Alphen, Samson et al. 2018), and pragmatic competence and prosociality have been shown to predict peer problems (Conti‐Ramsden et al. 2019). Other findings show that the role of pragmatics in predicting SEF is more prominent than the role of core language measures and that good pragmatic skills may even protect children with DLD from problems in SEF. For example, Ketelaars et al. (2010) showed that children with DLD with pragmatic difficulties exhibit more hyperactivity and have more emotional, conduct, and peer problems. Their deficits in structural language did not predict their problems in SEF once pragmatic difficulties were accounted for. Similar results were found by Van den Bedem, Dockrell, van Alphen, Kalicharan, and Rieffe (2018b), who showed that lower pragmatic skills were associated with more victimization in adolescence. Problems in syntactic ability, considered a core feature of DLD (Leonard 2014), did not contribute significantly. Good pragmatic skills and, relatedly, prosociality may in turn serve as a protective factor in SEF. Findings by Toseeb et al. (2020) indicate that high levels of prosociality in early childhood of children with DLD lead to fewer problems in SEF in middle childhood. Children with DLD who are more prosocial and who have good pragmatic skills have fewer behavioural and emotional problems (Mok et al. 2014).

Pragmatics has also been demonstrated to be related to QoL in young adults with learning disorders (Camia et al. 2022) and in children who are deaf or hard of hearing (Ching et al. 2021). To our knowledge, no studies have reported relations between communicative participation and QoL or pragmatics and QoL in children with DLD. However, most of the studies investigating QoL in DLD report the most serious problems in subdomains relating to SEF (Feeney et al. 2012). And some of the aforementioned SEF constructs, such as victimization, are beyond doubt connected to QoL. It is therefore deemed likely that relations between communicative participation and QoL may exist as well in DLD.

Based on the studies described above, a mediation model seems to arise: SEF and QoL in DLD are likely to be (partially) predicted by functional language measures such as pragmatics or communicative participation, rather than by structural language abilities alone. The current study investigates the role of communicative participation as a potential mediator in the relation between language and SEF and QoL.

## 2 Methods

### 2.1 Participants

This current study is part of the Dutch longitudinal DLD study (DLDLD‐study, www.projecttaalinzicht.nl). In this study, 600 children with (presumed) DLD^1^ were recruited via early intervention groups for children with DLD, located in different regions in the Netherlands.

All children in these early intervention groups went through the same standardized multidisciplinary diagnostic process at speech and hearing centres (Wiefferink et al. 2020). A diagnosis (presumed) of DLD is provided when scores on tests for language development fall at least 1.5 SD (standard deviations) below the norm in one or more language domains. Other diagnostic criteria are a nonverbal IQ of 70 or higher, no hearing loss, and no other biomedical condition that can explain the language difficulties.^2^ Referral to early intervention groups is based on the severity of the language difficulties and their functional impact in daily life. Exclusionary criteria for the present study were limited to parents’ (or primary caregivers’) ability to fill out Dutch questionnaires by themselves or with help from family or friends. At the start of the study, we gained informed consent from all parents of the recruited children.

The current study reports on the data of 511 children with data at two time points (T0 and T1), approximately 6–9 months apart. For 89 children, none of the questionnaires was completed at T1. Of the 511 children, 379 (74.2%) were boys and 132 (25.8%) were girls. Children had a mean age of 56.8 (SD = 3.11) months at T1. We measured socio‐economic status (SES) based on the education level of the least educated parent. For 52.8% of children, SES was high, that is, the least educated parent received a university or college degree. For a small portion of the children (7.6%), SES was low, meaning the least educated parent completed primary or secondary education. The remaining 38% had a middle SES, where the least educated parent completed vocational education. Table 1 presents characteristics of the children. In the context of this article, the term ‘parents’ includes ‘primary caregivers’.

**TABLE 1:** Means (SD) of raw scores and Pearson's correlations (two‐tailed).

	1.	2.	3.	4.	5.	6.	7.
	Age T1	LCQ	WQ	SQ	CP	SDQ	KINDL‐R
Mean	56.8	88.01	84.54	77.61	43.85	11.61	76.58
*SD*	*3.11*	*14.34*	*18.71*	*9.27*	*12.42*	*5.27*	*9.11*
1. Age T1	1						
2. LCQ	−0.22^**^	1					
3. WQ	−0.20^**^	0.59^**^	1				
4. SQ	−0.21^**^	0.48^**^	0.71^**^	1			
5. CP	−0.08	0.19^**^	0.31^**^	0.37^**^	1		
6. SDQ	0.01	−0.10^*^	−0.06	−0.04	−0.39^**^	1	
7. KINDL‐R	0.00	−0.04	0.02	0.04	0.47^**^	−0.63^**^	1

*Note*: ^*^ *p* < 0.05, ^**^ *p* < 0.01.

CP = communicative participation, KINDL–R = Quality of Life questionnaire, LCQ = language comprehension quotient, SDQ = Strengths & Difficulties Questionnaire, SQ = sentence quotient, WQ = word quotient.

### 2.2 Measures

#### 2.2.1 Language Ability

Language comprehension was tested with the Schlichting Test for Language Comprehension (Schlichting and Lutje Spelberg 2010a). Tasks included identifying the correct object to a spoken word, sentence comprehension, and executing tasks with test objects. Raw scores were transformed to quotient scores (language comprehension quotient—LCQ). Language production was tested with the Schlichting Test for Language Production (Schlichting and Lutje Spelberg 2010b), using subtests for expressive vocabulary (an object and picture naming task) and expressive grammar (a sentence elicitation task). Raw scores were transformed to quotient scores (word quotient [WQ] and sentence quotient [SQ], respectively). The quotient scores have a mean of 100 (SD = 15; normal range = 85–115). All tests have norms for children aged 2–7 years based on a representative sample of Dutch children. For all language tests, construct validity is good. Reliability measured through Lambda‐2's coefficient is good (averages for tests for Comprehension and Production 0.93 and 0.87, respectively; Schlichting and Lutje Spelberg 2010a, 2010b).

#### 2.2.2 SEF

Parents completed the Dutch version of the Strengths and Difficulties Questionnaire (SDQ; Goodman 1997, Dutch version: Van Widenfelt et al. 2003) to measure SEF. The SDQ determines children's strengths, psychosocial problems, and the influence of these problems on daily functioning (Goodman 1997). This questionnaire consists of 25 items, divided into five subscales:

- Hyperactivity/attention deficit.
- Emotional problems.
- Problems with peers.
- Behavioural problems.
- Prosocial behaviour.

Parents rated whether statements are not true (0 points), somewhat true (1 point) or certainly true (2 points). For data analysis, the total score of the SDQ was used. This is the sum of subscales one through four. Prosocial behaviour is not included in the total score because it represents positive behaviour rather than problem behaviour. The maximum score on the SDQ is 40 points, with a higher score reflecting more difficulties. Based on a Dutch norm group of children between 4 and 7 years of age (*n* = 1465), the total score can be denoted as normal (0–10 points), borderline (11–14 points) or raised (15 or more points; Theunissen et al. 2016). The test is norm‐referenced and has good construct validity. Internal consistency for the total score is sufficient (Cronbach's alpha = 0.79 in age group 4–6 years; Maurice‐Stam et al. 2018).

#### 2.2.3 QoL

To measure QoL, parents completed a Dutch translation of the KINDL‐R for children between 3 and 6 years of age (Kiddy‐KINDL_3‐6y; Ravens‐Sieberer and Bullinger 2000). This questionnaire consists of 24 statements about children's physical and emotional health, self‐esteem, family, friends, and school. Internal consistency is good (Cronbach's alpha = 0.82), and validity is satisfactory (Ravens‐Sieberer et al. 2007). The original German questionnaire was translated for our project following the publishers’ protocol. The scores on the KINDL‐R questionnaire range from 0 to 100, with higher scores indicating a higher QoL. Total scores can be compared with means and SDs in a sample of 3579 German children between 3 and 6 years old (mean total score = 80.04, SD *=* 8.12; Ravens‐Sieberer et al. 2007).

#### 2.2.4 Communicative Participation

The questionnaire for communicative participation consisted of nine questions concerning different communicative contexts (i.e., communication with parents, siblings, peers, and familiar and unfamiliar adults). Parents rated their child's communicative participation in these different communicative contexts on a 10‐point scale. A question is, for example, ‘My child is able to communicate independently with their siblings’. The questionnaire is an extension of a short questionnaire about parents’ perceptions of their child's functioning in communicative situations used by Zwitserlood et al. (2023). A full questionnaire used in this study can be found in the supplementary materials. Two of the nine questions were removed from analyses because of low internal consistency. The answers to the other seven questions are combined into one score for communicative participation (CP), with a maximum of 70. The reliability was good with a Cronbach's alpha of 0.88. Construct validity of this questionnaire has not been measured.

### 2.3 Measurement Procedure

Children's language abilities (i.e., LCQ, WQ, and SQ) were tested by a speech–language therapist or clinical linguist in the early intervention treatment groups. The final scores obtained in this setting were taken as starting point for this longitudinal study (T0).

Around 6–9 months later (T1), parents received the SDQ, KINDL‐R, and CP questionnaires via an online routine outcome monitoring (ROM) platform. Data were considered missing if parents did not fill out any of the questionnaires at T1 after two reminders via email and three phone calls.

### 2.4 Data Analysis

To answer our research question, path models were assessed using structural equation modelling with maximum likelihood estimation (in R 4.1.1, lavaan 0.6–9) with the language measures (LCQ, WQ, and SQ), the measure for communicative participation (CP), and the measures for well‐being (SDQ and KINDL‐R) as outcome measures. Missing data on outcome measures were imputed and outliers were checked and winsorized. LCQ had one outlier, as identified by standardized values below −3 or above +3 and incongruence with the normal distribution of the variable. The outlier was changed into the next highest value that was not an extreme outlier to prevent disproportioned influences on the results (Winsorizing; see Tabachnick and Fidell 2013: 11 for more information on this method). Two children were excluded because they were outliers on ‘age at T1’ and winsorization is not suitable for this variable. Seven children were outliers on ‘the time between T0 and T1’. Results for analyses with and without outliers were the same. Therefore, data including these seven children were used. All variables were normally distributed, and all other assumptions, such as linearity and independence of residuals, were met. To account for higher correlations between LCQ, WQ, and SQ, these variables were mean‐centred. Correlations between the predictor variables, *r* = 0.48–0.71, and variance inflation factors (VIFs), 1.17–2.41, indicated moderate correlations without causing problems with multicollinearity in the model.

**FIGURE 1:**
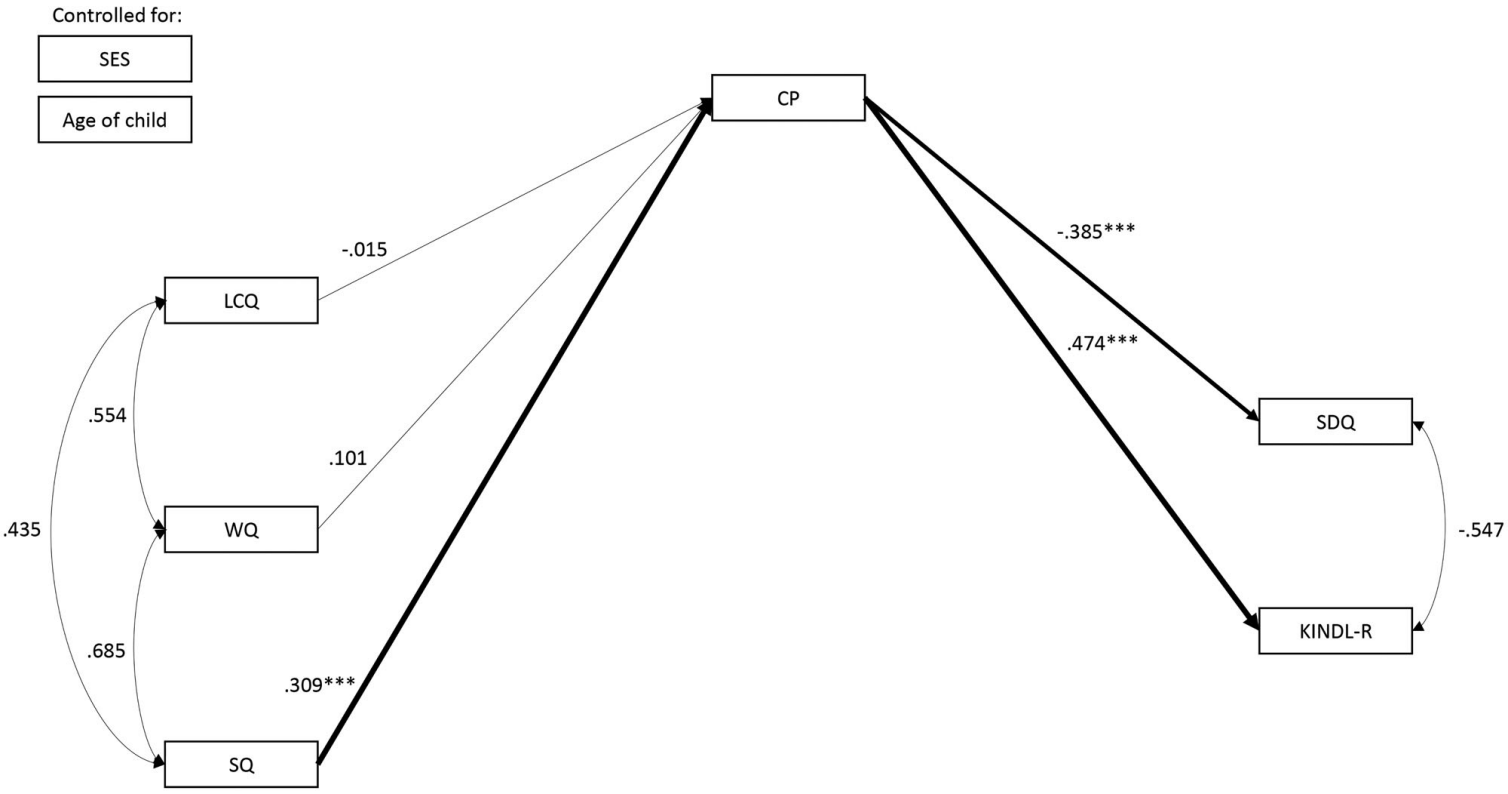
Standardized coefficients: Model 1: Indirect relations between language and SEF and QoL via communicative participation.

**FIGURE 2:**
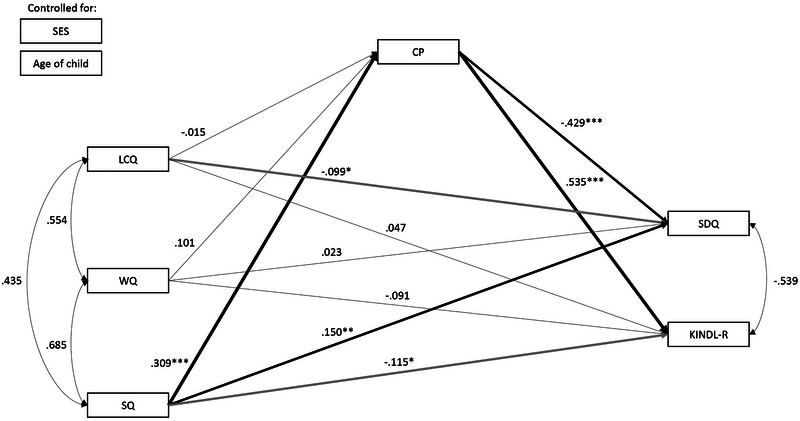
Standardized coefficients: Model 2: Relations between language and SEF and QoL, direct, and via communicative participation.

Model testing started with the theoretical model as shown in Figure 1. The following pathways were modelled: direct effects from language measures (LCQ, WQ, and SQ) to communicative participation (CP) and direct effects from communicative participation (CP) to both measures of well‐being (SDQ and KINDL‐R). Relations between LCQ, WQ, and SQ and between SDQ and KINDL‐R were modelled through covariances. The six total indirect effects through CP were also assessed. The model in Figure 2 was extended with direct effects from LCQ, WQ, and SQ to SDQ and KINDL‐R to assess full or partial mediation (Hayes 2009; Zhao et al. 2010).

The goodness of fit of the two models was assessed with chi‐square, CFI, and RMSEA. The focus of our analyses was on the regression coefficients of all pathways to indicate directions and strengths of the relations. Indirect relations were assessed using the Delta method (Raykov and Marcoulides 2004). The following statistics were reported: b, beta, standard error, confidence interval, *p*‐value, and explained variance. The significance of all relations was investigated with a significance level of 0.05. To decrease the false discovery rate due to multiple testing, the Benjamini Hochberg correction was used with a false discovery rate of 0.1 (Thissen et al. 2002).

## 3 Results

### 3.1 Missing Values

In total, 511 children were included in the analyses. All children had data for one or more language measures at T0 and were only included when one or more questionnaires of T1 were completed. Data were missing due to tests not being administered in the intervention groups or due to unreturned online questionnaires by parents. Little's MCAR test demonstrated that data were missing completely at random (*χ* ^2^(57) = 62.68, *p* = 0.282). To account for missing data, multiple imputation was executed by predictive mean matching (*k* = 5, number of imputations = 10). The final dataset was realized by calculating the mean of 10 imputations.

### 3.2 Descriptive Statistics

Correlations between the constructs and descriptive statistics on children's characteristics in the present study are reported in Table 1.

#### 3.2.1 Means and SDs

Compared with norms, the average language scores of the children in the present study fell within the 21st percentile on language comprehension (LCQ), 14th percentile on expressive vocabulary (WQ), and within the 6th percentile on expressive grammar (SQ). Expressive grammar seemed to be the weakest language ability within this sample; only 6% of peers in the norm group performed lower than the children in the present study on average.

In comparison with the Dutch norm data, the average score on the SDQ represented a borderline level of social–emotional problems. The majority of children obtained borderline scores (27.2%) or raised scores (26.4%), although a substantial percentage (46.4%) obtained a normal score. The average score on the KINDL‐R questionnaire for QoL was slightly lower than the German typically developing reference group (−0.43 SD). A substantial 30% of children scored more than −1 SD below the norm on this measure of QoL, compared with 16% in the reference group.

#### 3.2.2 Correlations

Small to moderate negative correlations existed between age at T1 and the language scores (LCQ, WQ, and SQ at T0, all *p* < 0.01). Mean language scores also differed between SES levels (one‐way analysis of variance (ANOVA), all *p* < 0.01). Because of the significant relations with language scores, age at T1 and SES were added to the path analyses as covariates.

All language quotient scores showed significant positive correlations with communicative participation (CP) as well, some even relatively strong (all *p* < 0.01). The measure of SEF (SDQ) showed significant negative correlations with language comprehension (LCQ, *p* < 0.05) and communicative participation (CP) (*p* < 0.01). These correlations were small to moderate. Lastly, QoL (as measured with the KINDL‐R) showed a significant positive correlation with communicative participation (CP) and a negative correlation with SEF (measured with the SDQ) (both *p* < 0.01 and a moderate and strong effect size, respectively).

All language scores (LCQ, SQ, and WQ) related significantly and positively to each other, with moderate to strong effect sizes. Despite the significance, none of these correlations exceeded a value of *r* = 0.80, indicating that all tests seemed to measure different constructs.

### 3.3 Path Analyses

A visual representation of model 1 and the standardized coefficients are reported in Figure 1. Unstandardized coefficients are reported in Table 2. The model, investigating the indirect relations between language and SEF and QoL through communicative participation, adequately fits the data, *χ* ^2^(12) = 34.70, *p* = 0.001, CFI = 0.98, RMSEA = 0.06. All relations are examined whilst controlling for SES and age of the child. Coefficients for the relations between SES, age, and predictor variables are reported in Table 3. The total indirect effect of SQ on SDQ via CP was a significant negative effect, indicating that children with higher expressive grammar have fewer behavioural problems as reported by their parents. This is a small effect. Further inspection of the indirect relation showed a positive and significant direct effect from SQ to CP, indicating that parents reported a higher communicative participation for children with higher expressive grammar. This was a moderate effect. Higher CP, in turn, related to lower SDQ (fewer problems), with a moderate effect size.

**TABLE 2:** Path coefficients: Model 1.

Covariances			*b*	SE	Beta	*p*‐value
LCQ	<–>	WQ	0.51	0.05	0.55	< 0.001^***^
WQ	<–>	SQ	0.64	0.05	0.69	< 0.001^***^
LCQ	<–>	SQ	0.40	0.05	0.44	< 0.001^***^
SDQ	<–>	KINDL‐R	−20.51	1.89	−0.55	< 0.001^***^
**Indirect relations**						
LCQ	To	CP	−0.19	0.63	−0.02	0.765^a^
WQ	To	CP	1.25	0.79	0.10	0.112^a^
SQ	To	CP	3.83	0.72	0.31	< 0.001^[Link]^, ^[Link]^
CP	To	SDQ	−0.16	0.02	−0.39	< 0.001^[Link]^, ^[Link]^
CP	To	KINDL‐R	0.33	0.03	0.47	< 0.001^[Link]^, ^[Link]^
**Total indirect relations**						
LCQ	to CP to	SDQ	0.03	0.10	0.01	0.765^a^
LCQ	to CP to	KINDL‐R	−0.06	0.21	−0.01	0.765^a^
WQ	to CP to	SDQ	−0.21	0.13	−0.04	0.117^a^
WQ	to CP to	KINDL‐R	0.42	0.27	0.05	0.115^a^
SQ	to CP to	SDQ	−0.63	0.14	−0.12	< 0.001^[Link]^, ^[Link]^
SQ	to CP to	KINDL‐R	1.28	0.26	0.15	< 0.001^[Link]^, ^[Link]^

*Note*: ^*^ *p* < 0.05; ^**^ *p* < 0.01; ^***^ *p* < 0.001.

^a^Regressions corrected for multiple testing with the Benjamini–Hochberg correction (FDR = 0.1).

^b^No longer significant after correction for multiple testing with the Benjamini–Hochberg correction (FDR = 0.1).

CP = communicative participation, KINDL‐R = KINDL‐R Quality of Life questionnaire, LCQ = language comprehension quotient, SDQ = Strengths & Difficulties Questionnaire, SQ = sentence quotient, WQ = word quotient.

**TABLE 3:** Coefficients between covariates (SES and age of child) and predictor variables (LCQ, WQ and SQ).

Covariances			*b*	SE	Beta	*p*‐value
SES	<–>	Age of child	−0.07	0.09	−0.03	0.443
**Indirect relations **						
SES	to	LCQ	0.30	0.07	0.19	< 0.001^***^
Age of child	to	LCQ	−0.07	0.01	−0.21	< 0.001^***^
SES	to	WQ	0.32	0.07	0.20	< 0.001^***^
Age of child	to	WQ	−0.06	0.01	−0.19	< 0.001^***^
SES	to	SQ	0.22	0.07	0.14	0.001^**^
Age of child	to	SQ	−0.07	0.01	−0.20	< 0.001^***^

*Note*: ^*^ *p* < 0.05; ^**^ *p* < 0.01; ^***^ *p* < 0.001.

LCQ = language comprehension quotient, SES = social economic status, SQ = sentence quotient, WQ = word quotient.

The total indirect effect of SQ on KINDL‐R via CP was a significant positive effect, indicating that children with higher expressive grammar have higher QoL reported by their parents. This was a small effect. Further inspection of the relation between CP and KINDL‐R showed a significant moderate effect. Children whose parents reported a higher communicative participation also had a higher reported QoL.

SDQ and KINDL‐R related significantly and negatively to each other, indicating that children with fewer social–emotional problems had higher QoL scores. This was a small effect. LCQ and WQ did not relate significantly to CP, nor indirectly to SDQ and KINDL‐R via CP, indicating that language comprehension and expressive vocabulary do not contribute significantly to the explained variance in well‐being beyond expressive grammar. In this model, 14.3% of the total variance in CP is explained by the language scores. For SDQ and KINDL‐R, 14.8% and 22.4% of the variance are explained, respectively.

A visual representation of model 2 and the standardized coefficients are reported in Figure 2. Unstandardized coefficients are reported in Table 4. The model, investigating both direct and indirect relations, closely fits the data, *χ* ^2^(6) = 13.05, *p* = 0.042, CFI = 0.994, RMSEA = 0.05. The second model, also including the direct pathways, fits the data significantly better than the model only including indirect pathways, Δ*χ* ^2^ = 21.66, *p* = 0.001. This follows naturally from the decrease in degrees of freedom.

**TABLE 4:** Path coefficients: Model 2.

Covariances			*b*	SE	Beta	*p*‐value
LCQ	<–>	WQ	0.51	0.05	0.55	< 0.001^***^
WQ	<–>	SQ	0.64	0.05	0.69	< 0.001^***^
LCQ	<–>	SQ	0.40	0.05	0.44	< 0.001^***^
SDQ	<–>	KINDL‐R	−19.69	1.84	−0.54	< 0.001^***^
**Indirect relations**						
LCQ	to	CP	−0.19	0.63	−0.02	0.765^a^
WQ	to	CP	1.25	0.79	0.10	0.112^a^
SQ	to	CP	3.83	0.72	0.31	< 0.001^[Link]^, ^[Link]^
CP	to	SDQ	−0.18	0.02	−0.43	< 0.001^[Link]^, ^[Link]^
CP	to	KINDL‐R	0.38	0.03	0.54	< 0.001^[Link]^, ^[Link]^
**Total indirect relations**						
LCQ	to CP to	SDQ	0.04	0.12	0.01	0.765^a^
LCQ	to CP to	KINDL‐R	−0.07	0.24	−0.01	0.765^a^
WQ	to CP to	SDQ	−0.23	0.15	−0.04	0.116^a^
WQ	to CP to	KINDL‐R	0.47	0.30	0.05	0.115^a^
SQ	to CP to	SDQ	−0.70	0.15	−0.13	< 0.001^[Link]^, ^[Link]^
SQ	to CP to	KINDL‐R	1.45	0.30	0.17	< 0.001^[Link]^, ^[Link]^
**Direct relations**						
LCQ	to	SDQ	−0.52	0.27	−0.10	0.050^[Link]^, ^[Link]^
LCQ	to	KINDL‐R	0.41	0.42	0.05	0.329^a^
WQ	to	SDQ	0.12	0.33	0.02	0.718^a^
WQ	to	KINDL‐R	−0.79	0.52	−0.09	0.128^a^
SQ	to	SDQ	0.79	0.31	0.15	0.011^[Link]^, ^[Link]^
SQ	to	KINDL‐R	−1.00	0.49	−0.12	0.040^[Link]^, ^[Link]^
**Total relations**						
LCQ	to CP to	SDQ	−0.49	0.29	−0.09	0.093
LCQ	to CP to	KINDL‐R	0.34	0.48	0.04	0.484
WQ	to CP to	SDQ	−0.11	0.36	−0.02	0.762
WQ	to CP to	KINDL‐R	−0.32	0.60	−0.04	0.592
SQ	to CP to	SDQ	0.09	0.33	0.02	0.784
SQ	to CP to	KINDL‐R	0.44	0.55	0.05	0.421

*Note*: ^*^ *p* < 0.05; ^**^ *p* < 0.01; ^***^ *p* < 0.001.

^a^Regressions corrected for multiple testing with the Benjamini–Hochberg correction (FDR = 0.1).

^b^No longer significant after correction for multiple testing with the Benjamini–Hochberg correction (FDR = 0.1).

CP = communicative participation, KINDL‐R = KINDL‐R Quality of Life questionnaire, LCQ = Language Comprehension Quotient, SDQ = Strengths & Difficulties Questionnaire, SQ = sentence quotient, WQ = word quotient.

In line with model 1, in model 2 language comprehension and expressive vocabulary (LCQ and WQ) still do not relate significantly to the measures of well‐being (SDQ and KINDL‐R), neither directly nor indirectly. The weak negative relation between LCQ and SDQ, which is shown in Figure 2, was no longer significant after correcting for multiple testing. The significant total indirect effects from expressive grammar (SQ) to well‐being (SDQ and KINDL‐R), via communicative participation (CP), also remain similar to model 1. By including the direct effects between the language scores and SDQ and KINDL‐R, the explained variance of SDQ increased to 16.7% (+1.9%) and the explained variance of KINDL‐R increased to 24.9% (+2.5%). Model 2 thus explained more variance in both measures of well‐being. All total effects were nonsignificant.

By including the direct effects from the language scores on SDQ and KINDL‐R, it was found that SQ also related significantly to SDQ and KINDL‐R directly, with a small effect size. Both relations seem to indicate an unexpected direct negative relationship from expressive grammar to well‐being. The weak negative relation between SQ and KINDL‐R, which is shown in Figure 2, was no longer significant after correcting for multiple testing. The positive effect between SQ and SDQ did remain significant. As a higher score on SDQ reflects more problems, it seems to indicate that children with higher expressive grammar scores have more social–emotional problems. This is in contrast with the total indirect effect being negative and with the hypotheses based on the literature. Moreover, the correlation between SQ and SDQ was −0.04, thus not indicating a positive significant relation either. Considering these unexpected relations, CP seemed to have a suppressing effect on the relation between SQ and well‐being. This might explain why the total effects (from SQ to well‐being via CP and directly) are nonsignificant. To investigate this phenomenon, post‐hoc analyses were executed.

### 3.4 Post‐hoc Analyses

The significant and positive direct relation between expressive grammar (SQ) and SEF (SDQ) in model 2 was unexpected based on their nonsignificant correlation (*r* = −0.04) and the hypothesis based on the literature. The inclusion of communicative participation in the model seemed to influence the direct relation between these domains. To explore this more thoroughly, we investigated SDQ scores and the relation between SQ and SDQ at different levels of communicative participation. First, we divided children into two groups based on their expressive grammar, that is, children with below‐average SQ scores (< 85) and children with average SQ scores (≥ 85). Children were also divided into two groups based on their communicative participation (CP) scores. In the absence of norm scores, we chose to divide children into the 50% with the lowest CP scores and the 50% with the highest CP scores. This resulted in four different groups: children with (1) lower SQ and lower CP, (2) lower SQ and higher CP, (3) higher SQ but lower CP, and (4) higher SQ and higher CP. The SDQ score distribution in ‘normal scores’ (0–10), ‘borderline scores’ (11–14), and ‘raised scores’ (> 15) was investigated. For these four groups, a chi‐square difference test confirmed differences between the groups in SDQ score distribution, *χ* ^2^(6) = 64.95, *p* < 0.001 (Table 5).

**TABLE 5:** Post‐hoc analyses SDQ scores.

Groups	Normal, *n* (%)	Borderline, *n* (%)	Raised, *n* (%)	Total, *N*	Correlation SQ and SDQ	*p*‐value
Lower CP and lower SQ	74 (33.2%)	66 (29.6%)	83 (37.2%)	223	*r* = 0.076	0.260
Lower CP and higher SQ	6 (20%)	7 (23.3%)	17 (56.7%)	30	*r* = 0.381^*^	0.038
Higher CP and lower SQ	98 (57.0%)	48 (27.9%)	26 (15.1%)	172	*r* = 0.038	0.621
Higher CP and higher SQ	59 (68.6%)	18 (20.9%)	9 (10.5%)	86	*r* = −0.026	0.813

*Note*: ^*^ *p* < 0.05; ^**^ *p* < 0.01.

CP = communicative participation, SQ = sentence quotient.

Children who scored lower on CP more often had borderline or raised SDQ scores than children with higher CP (67–80% for the two groups with lower CP and 31–43% for the two groups with higher CP). This is in line with the significant relations between communicative participation and SEF. The chance of social–emotional problems was specifically high for children with lower CP but higher SQ: borderline and raised SDQ scores were reported for 80% of the children. This was also represented when investigating the correlation between SQ and SDQ for these four groups separately. Only for children with lower CP and higher SQ, a significant and positive correlation between SQ and SDQ was found, *p* = 0.038 (Table 5). In the group of children with low communicative participation but high expressive grammar skills, expressive grammar thus seems negatively correlated with SEF. This small subgroup might therefore be responsible for the statistical suppressor effect we found in the analyses of model 2.

## 4 Discussion

This study investigated the role of communicative participation as a potential mediator in the relation between language, SEF (measured by the SDQ), and QoL (measured by the KINDL‐R) in children with DLD. Based on previous findings that functional language measures such as pragmatic skills account for more variance in SEF than structural language measures (Ketelaars et al. 2010; Van den Bedem, Dockrell, van Alphen, Kalicharan, and Rieffe 2018), we hypothesized a mediating role for communicative participation. The results showed that communicative participation indeed mediates the relation between language and both domains of well‐being. Children's language scores were positively related to their communicative participation and well‐being according to parents. Expressive grammatical ability in particular predicted QoL and SEF via communicative participation. Language comprehension and expressive vocabulary did not appear to explain any variance in communicative participation, SEF or QoL on top of expressive grammar.

We tested two models in this study, one excluding direct relations between language (model 1), SEF, and QoL, and one including direct relations (model 2). When adding direct relations to the model, the model improved and indirect relations from language via communicative participation to SEF and QoL remained significant.

Interestingly, model 2 found an unexpected direct relation between expressive grammar and SEF, indicating that better expressive grammatical skills were related to more social–emotional problems (a similar unexpected negative relation was found with QoL, but this relation was not significant after correcting for multiple tests). This contrasted strongly with the absence of a correlation between these two concepts and the total indirect relation. Based on the literature, better language abilities are also expected to be related to fewer instead of more problems in SEF (e.g., Goh et al. 2021). Post hoc analyses showed that this unexpected relation could likely be explained by a small subgroup of children with strong expressive grammar but weak communicative participation. The unexpected relation between expressive grammar and SEF was found only in this particular subgroup. For this subgroup, grammatical skills in standardized tests might not reflect their capacity in daily communication, possibly resulting in frustration or negative emotions and parent‐reported problems in SEF. We assume that this group might have caused communicative participation to act as a statistical suppressor on the relation between expressive grammar and SEF in the second model, causing the unexpected relations between expressive grammar and well‐being and non‐significant total effects in the total group. This implies that, in clinical practice, we also need to look carefully at sub‐profiles and that relationships do not always have the expected direction. Taken together, however, the results indicate that communicative participation plays a major mediating role in the relation between language, SEF, and QoL.

The finding that children with DLD have problems in SEF is in line with previous studies (Chen et al. 2020; Goh et al. 2021; Van den Bedem, Dockrell, van Alphen, Samson et al. 2018; Van den Bedem, Dockrell, van Alphen, Kalicharan, and Rieffe 2018). As in other studies on SEF, scores in our study showed a large variation. Though the majority of children in our study obtained borderline or raised scores, there also was a substantial percentage that obtained a normal score, emphasizing the heterogeneity of children with DLD. The prevalence of behaviour problems might increase with age (Curtis et al. 2018). This study found language skills and communicative participation to explain only part of the variance in parent‐reported SEF of young children with DLD (16.7%). This indicates that there are other predictive factors influencing well‐being in children with DLD.

In our study, QoL scores were on average slightly lower than scores from a sample of typically developing German children of the same age (Ravens‐Sieberer et al. 2007). Previous work that showed QoL to be affected in children and adolescents with DLD (e.g., Eadie et al. 2018; Le et al. 2021) also indicated that differences between children with language difficulties and typical development are smaller or nonsignificant at younger ages and increase with age. This either reflects that over time QoL is declining or that parents gain more insight into the well‐being of their children. Although QoL scores were on average not drastically lower, there is a large amount of variation in individual scores, where many children (30%) in our sample obtained scores below −1 SD. A total of 25% of the variation in QoL can be explained by communicative participation and language scores. As stated above, this leaves room for other factors to explain variation in well‐being (Eadie et al. 2018).

It is important for practitioners to be aware of the variation in well‐being in this group of children and the role that functional measures of language, such as communicative participation, play in this variation. This emphasizes the need to focus on children's interactions with other people. This idea is corroborated by findings from Forrest et al. (2021), who looked into the relation between DLD status and social–emotional problems as reported by parents and showed that peer relations mediated the relation between language status and social–emotional problems (Forrest et al. 2021). Treatment and educational support for children with DLD should therefore aim at enhancing children's communicative skills and social circumstances. Including more functional measures of language ability in clinical practice and in research on outcomes of language problems in children with DLD is therefore pivotal and in line with recommendations by the CATALISE consortium that multiple sources of information should be combined when assessing children (Bishop et al. 2016).

The children enrolled in our study were diagnosed with (presumed) DLD at a young age, indicating they had difficulties acquiring language that may persist as they grow older. On average, the results of our study showed that children had weaker language skills when they entered school. This was especially true for expressive grammar. Children also experienced problems in expressive vocabulary, though to a lesser extent. Language comprehension was least affected. These observations are in line with the literature on DLD, showing that many children with DLD struggle with expressive grammar, while their comprehension is often average (Bruinsma et al. 2022; Leonard 2014; Vermeij et al. 2021). Expressive grammatical problems thus seem to be a core deficit in DLD and have been found to be a sensitive predictor for outcomes such as school placement or reading comprehension (Duinmeijer et al., 2021; Muter et al. 2004). Our results show that deficits in expressive grammar in particular seemed to affect well‐being, where children with higher expressive grammar scores had better communicative participation and experienced fewer social–emotional problems. Recently, Verbeek et al. (2023) reported a similar link between expressive grammar and SEF. Since we follow children longitudinally in language, communicative participation, and well‐being, we will be able to investigate the relationship between these domains more thoroughly in the future.

This study also has a few limitations. Since we are only following a group of children with (presumed) DLD, we do not have a control group to compare scores to. Most of our tests are norm‐referenced, but for communicative participation, normed scores are not available, which makes it more difficult to interpret children's scores. Since the start of our data collection, other instruments to measure communicative participation have been developed that are currently more widely used than the questionnaire we included in our study. For example, a parental questionnaire suitable for children is the FOCUS‐34 (Cunningham et al. 2020), a shortened version of the original FOCUS questionnaire with 50 questions. This questionnaire has good reliability and validity and has been translated into several languages. In future research and clinical practice, using instruments such as the FOCUS‐34 or other suitable (normed) instruments should be taken into consideration.

It should also be noted that on average, children came from families with a relatively high SES. Although this is a common limitation in research on DLD, it means that our results cannot be generalized to children from families with a lower SES.

Finally, while the constructs of communicative participation, SEF, and QoL can statistically be distinguished as separate constructs, theoretically the scales within the various questionnaires seem to overlap. For example, the SDQ questionnaire subscale *‘peer relations’* seems to focus on communicative aspects of the relation with peers. It is not unlikely that this, to some extent, also measures communicative participation. Similarly, the KINDL questionnaire has a subscale *‘social relations’* with questions that resemble the questions of the ‘peer relations’ subscale of the SDQ. Future research endeavours could focus on disentangling the domains of SEF, QoL, and communicative participation to ensure higher construct validity.

## 5 Conclusions

In summary, the finding that communicative participation mediates the relation between language and well‐being underlines the importance of addressing communicative participation both in research on and in clinical practice for children with DLD. Our results indicate that communicative participation—as a more functional measure of language ability—can provide added value when investigating relations between language and other developmental domains in children with DLD, on top of measuring core language skills (Cunningham et al. 2019). For clinicians, these results may strengthen the view that treatment for DLD should (also) focus on the ability to participate in communication and society (Bishop et al. 2016; WHO 2001). Measuring communicative participation can aid clinicians in identifying a child's specific communicative needs and determining the most suitable setting for providing support. Measuring communicative participation may also help clinicians to form treatment plans, evaluate intervention effects and provide parents with advice. Furthermore, it is important for clinicians to be aware of the relations between language ability, communicative participation, and well‐being, given the lower scores children obtained on the latter domain. Taking into consideration the higher risk children with DLD have to develop psychiatric problems as they grow older (e.g., St. Clair et al. 2011), it may prove worthy to measure children's SEF and QoL in early treatment and school settings. Recognizing that early language difficulties can impact well‐being may constitute an initial step towards providing more effective support for children with DLD.

## Ethics Statement

Ethical approval was granted by the Dutch ETC‐SACB (reference number 23‐020‐01).

## Conflicts of Interest

The authors declare no conflicts of interest.

## Data Availability

The data that support the findings of this study are available from the corresponding author upon reasonable request.

## Endnotes

1 In the Netherlands, children can receive a DLD diagnosis relatively early due national screening procedures. For young children (at least up to 3 years of age), a label ‘presumed’ DLD is used. Since the criteria for this label are equal to the criteria for DLD, the term DLD will be used throughout this article.

2 In the Netherlands, after diagnostic assessment, children attend either individual speech–language therapy in private practices or multi‐component intervention in early intervention groups, that is, special language daycare centers. Treatment in early intervention groups is delivered by a multidisciplinary team with a psychologist, SLP and pedagogical assistants. Children visit the groups between 10 and 15 h each week. Treatment for the children consists of group activities with pedagogical assistants using language support strategies, and direct intervention by the SLP. During group activities, playtime alternates with, amongst other things, vocabulary intervention protocols, singing, interactive book reading and mealtimes. Three types of activities are distinguished: (1) daily routines, such as welcoming, eating and drinking; (2) educational play; and (3) intentional language stimulation, including dialogic picture‐book reading. Parents are offered group meetings in which they receive general information about language development, parents’ role in their child's language development, managing behavioral problems and social–emotional developmental problems. In these group meetings, parents can also share problems and discuss their experiences with their children in daily life.

## Appendix 1 Questionnaire for Communicative Participation

Question 1^3^

How severe is the limitation in communication of your child in comparison with peers?

Minimal limitation      1    2    3    4    5    6    7    8    9    10      Maximal limitation

*Question 2*

How worried are you about your child's speech–language development?

Not at all       1    2    3    4    5    6    7    8    9    10      Extremely worried

*Question 3*

My child is able to communicate independently^4^ with us, their parent(s)/caregiver(s).

Not at all       1    2    3    4    5    6    7    8    9    10      Very well

*Question 4*

My child is able to communicate independently with their siblings or other members of the immediate family.

Not at all       1    2    3    4    5    6    7    8    9    10      Very well

*Question 5*

My child is able to communicate independently with other children.

Not at all       1    2    3    4    5    6    7    8    9    10      Very well

*Question 6*

My child is able to communicate independently with familiar adults.

Not at all       1    2    3    4    5    6    7    8    9    10      Very well

*Question 7*

My child is able to communicate independently with unfamiliar adults.

Not at all       1    2    3    4    5    6    7    8    9    10      Very well

*Question 8*

My child has social interaction with children outside of school hours.

Not at all       1    2    3    4    5    6    7    8    9    10      Very often

*Question 9*

My child has friendships with children.

Not at all       1    2    3    4    5    6    7    8    9    10      Many
